# Scrotal Verrucous Carcinoma: An Exceptional Localization of a Rare Tumor

**DOI:** 10.1155/2021/6651925

**Published:** 2021-02-22

**Authors:** K. Imrani, A. Lahfidi, L. Belkouchi, H. Jerguigue, R. Latib, Y. Omor

**Affiliations:** Radiology Department, National Institute of Oncology, Mohammed V University, Rabat, Morocco

## Abstract

Scrotal verrucous carcinoma is a rare entity. It is rarely metastatic especially in lymph nodes. Imaging is important for local extension in order to guide the surgical procedure. The diagnostic is histological. The treatment is based on surgical excision. The prognosis is relatively good, but local recurrences are frequent. We report a case of scrotal verrucous carcinoma in a 49-year-old man evolving for 1 year.

## 1. Introduction

Verrucous carcinoma was first described in 1948 in the oral cavity. Scrotal localization is very rare. The diagnosis is confirmed histologically [1]. Magnetic resonance imaging provides local extension of the tumor in order to guide the surgical excision, which is the only curative treatment [2].

## 2. Case Report

**Figure 1:**
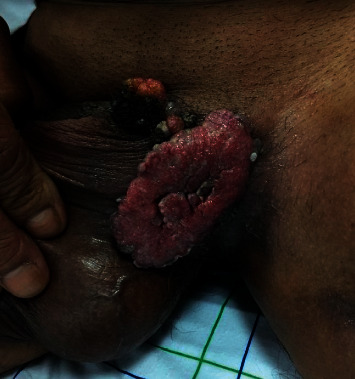
Inguinoscrotal exophytic lesion with a warty surface giving a cauliflower appearance.

The case was a 49-year-old man presenting to the emergency department for a vegetative inguinoscrotal lesion increasing in size for 1 year. On clinical examination, the lesion was purulent, painless, with well-defined irregular contours, and a warty surface, resembling a cauliflower, measuring 6 × 5 mm on the left and 4 × 3 cm on the right (Figure 1). There was bilateral inguinal lymphadenopathy.

**Figure 2:**
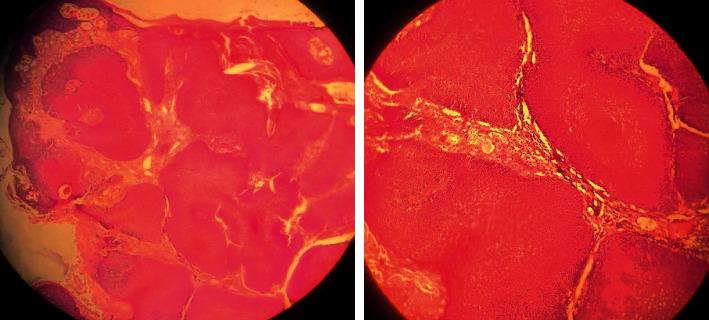
Pathological examination showing a very well-differentiated squamous cell tumor proliferation with an exophytic and endophytic tumor architecture bordered by hyperkeratosis and parakeratosic epidermis.

A biopsy of the tumor was performed, and the anatomopathological study showed a very well-differentiated squamous cell tumor proliferation with an exophytic and endophytic tumor architecture bordered by hyperkeratosis and parakeratosic epidermis in favor of a verrucous-type carcinoma (Figure 2).

**Figure 3:**
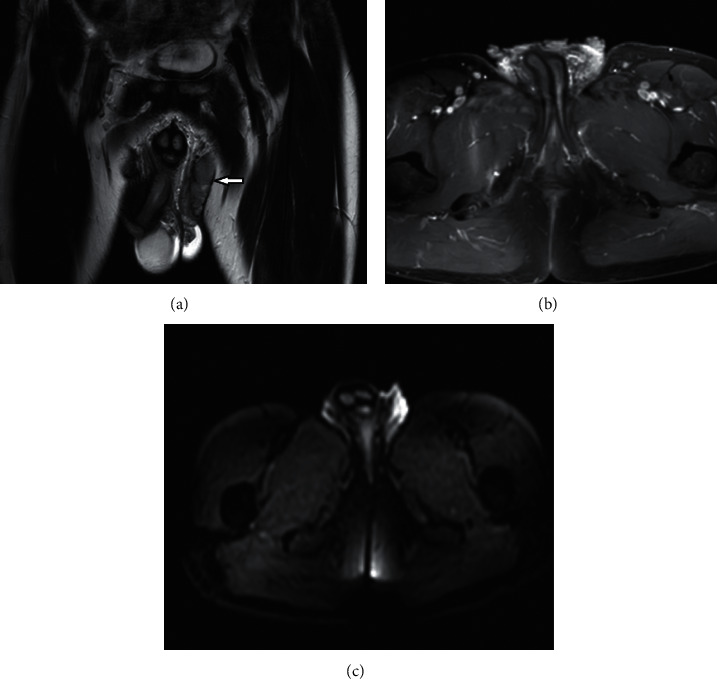
Inguinoscrotal MRI showing the exo- and endophytic inguinoscrotal tumor with intermediate signal intensity on T2-weighted sequences ((a), arrow) enhanced ((b), star) with diffusion restriction (c).

Inguinoscrotal MRI was performed for local invasion before surgery. It showed a bilateral exo- and endophytic inguinoscrotal tumor with intermediate signal intensity on T2-weighted images with contrast enhancement and restricted diffusion (Figure 3). The left epididymal head was invaded.

Surgical excision of the tumor with left orchidectomy was performed. Extemporaneous examination of inguinal lymphadenopathies confirmed their metastatic nature, followed by the inguinal lymph node dissection. The surgical wound was closed with a scrotal flap. Resection margins were negative according to the anatomopathological examination. At a follow-up 18 months later, the clinical examination was quite normal, and there was no tumor recurrence.

## 3. Discussion

Scrotal verrucous carcinoma is a rare entity, first described by Ackerman in 1948 as a variant of squamous cell carcinoma, characterized by exophytic growth without infiltration of the basal membrane with a locally invasive but rarely metastatic development, especially in lymph nodes [3, 4].

Human papillomaviruses (HPV) types 6 and 11 would have a significant role in the pathogenesis of verrucous carcinoma. However, some studies have not found an association between these tumors and HPV [3].

Clinically, the tumor has a budding, cauliflower appearance, sometimes associated with a superficial, foul-smelling ulceration.

The relationship between Buschke–Lowenstein tumor or giant acuminate condyloma and verrucous carcinoma remains unclear. Some authors consider them two progressive forms of the same disease, while others as separate diseases. Whatever the relationship between these two conditions, their treatment is identical, primarily requiring surgery. Verrucous carcinoma is not related to HPV infection. It is locally aggressive, with possible metastatic potential, and therefore has a poor prognosis. Lowenstein tumor has a low rate of recurrence which carries a good prognosis [4, 5].

The biopsy with anatomopathological study confirms the diagnosis of verrucous carcinoma, showing hyperplasia with hyperacanthosis, papillomatosis, and sometimes hyperkeratosis surface. Epithelial buds push back the underlying chorion, respecting the basement membrane. Imaging provides a local extension in order to guide the surgical procedure.

Ultrasound shows the tumor extension to the spermatic cord, epididymis, testicles, and penis. The lesion can be iso- or hypoechoic, well defined, with irregular contours, vascularized at color Doppler.

Magnetic resonance imaging is more sensitive for evaluating the locoregional extension of the tumor and for looking for lymphadenopathy. Scrotal verrucous carcinoma is hypointense on T1 WS, with intermediate signal intensity on T2 WS, with contrast enhancement and restricted diffusion.

Surgical excision with lymph node dissection (in case of associated metastatic lymph nodes) is the standard treatment; it must be wide enough to have negative resection margins [5]. The prognosis is relatively good, but local recurrences are frequent. This justifies clinical monitoring every 4 months during the first year, every 6 months for 2 years, and then every year with a clinical examination supplemented by ultrasound, fine needle aspiration, and screening smear [4].

## 4. Conclusion

Scrotal verrucous carcinoma is a rare mucosal skin disorder. The clinical appearance of the tumor is suggestive, but the positive diagnosis is based on a biopsy with histological study. Imaging is important for local and regional tumor extension. The treatment is based on surgical excision, which must be radical because of the course of frequent recurrences.
